# The Role of Diptera and Coleoptera (Arthropoda: Insecta) in Identifying Injuries and Estimating Postmortem Interval: A Case Report

**DOI:** 10.1007/s13744-026-01444-0

**Published:** 2026-09-28

**Authors:** Carina Mara Souza, Vinícius da Costa-Silva, Andre Gardelino Savino, Aline Marrara Prado, Patricia Jacqueline Thyssen

**Affiliations:** 1 Lab Integrative Entomology, Department of Animal Biology, Institute of Biology, Univ Estadual de Campinas (UNICAMP), Campinas, São Paulo State 13083-862 Brazil; 2 Campo Experimental Getúlio Vargas, Empresa de Pesquisa Agropecuária de Minas Gerais (EPAMIG/Oeste), Uberaba, Minas Gerais State Brazil; 3 Invertebrate Biosystematics and Conservation Group, Dept of Zoology & Entomology, Univ of Pretoria, Pretoria, South Africa; 4 Forensic Police of São Paulo State, Superintendência da Polícia Técnico-Científica, Jundiaí, São Paulo State, Brazil

**Keywords:** *Hemilucilia segmentaria*, *Sarcophaga* (*Lyopygia*) *ruficornis*, *Oxelytrum discicolle*, Necrophagous species, Postmortem artifacts, Forensic entomology

## Abstract

Diptera and Coleoptera (Arthropoda: Insecta) are of great importance in forensic entomology for providing valuable information that contribute to investigations of the cause of death, cases of neglect and mistreatment or for estimating post-mortem interval (PMI), among others. Considering that the period of insect activity (PIA) reliably and accurately represents the PMI and the increasing use of entomological evidence in forensic cases, we report a case in which *Hemilucilia segmentaria* (Fabricius) (Diptera, Calliphoridae) was used as entomological evidence to estimate the PMI in a suspected death of a woman whose body was found in a wild environment in southeastern Brazil. Additionally, we report the breeding of *Sarcophaga* (*Lyopygia*) *ruficornis* (Fabricius) (Diptera, Sarcophagidae) in the corpse, highlighting the need of data development of this species for accurately estimation of PMI, and *postmortem* lesions caused by the previous fly species and by the carrion beetle *Oxelytrum discicolle* (Brullé) (Coleoptera, Staphylinidae, Silphinae), emphasizing the importance of the knowledge about the biology and behaviour of necrophagous species in order to avoid misinterpretation of evidences.

## Introduction

The use of distribution, ecology, and biology information of necrophagous insects are valuable in forensic entomology application for legal purposes (Catts and Goff 1992; Lutz et al. 2021). These data contribute to the investigation of the cause of death, highlighting cases of neglect and mistreatment or for estimating post-mortem interval (PMI) (e.g., Thyssen et al. 2012, 2018).

PMI can be estimated based on the period of insect activity (PIA), which may be evaluated through different methodological approaches (see Kashyap and Pillai 1989; Amendt et al. 2007). One of them involves estimating the age of collected immature insects using morphometric parameters, as larval length and/or body mass, combined with temperature data from where the corpse was found (e.g., Thyssen et al. 2012, 2018; Matuszewski 2021; Thyssen and Oliveira-Costa 2024). Alternatively, PIA estimation can be achieved through linear temperature-dependent developmental models that incorporate species-specific developmental rates and accumulated thermal requirements (Ikemoto and Takai 2000; Amendt et al. 2007). Furthermore, after the emergence and dispersal of the first insect generation, PMI estimation may also rely on ecological succession patterns, considering the chronological colonization of insect taxa associated with distinct stages of decomposition (Amendt et al. 2007; Lefebvre and Gaudry 2009; Matuszewski 2021).

Activity of insects and other arthropods can also be useful in reconstructing *perimortem* events; however, when evaluating a crime scene and also thanatological and medico-legal information of a corpse, it is important to consider that insect activity can produce *postmortem* artifacts, ranging from modifications in bloodstain patterns to lesions caused by their feeding behavior on the decomposing tissues. These artifacts can be misinterpreted as *antemortem* events and lesions, linked with the cause of death, leading to erroneous understanding of the facts that ended in the death (Viero et al. 2019).

It is important to emphasize that reliability of information retrieved from entomological evidences depends on a previous and accurate identification of the associated entomofauna, whether through morphological, molecular, chemical, or integrative approaches (e.g., Thyssen 2005; Thyssen et al. 2005; Madeira et al. 2016; Prado et al. 2023; Grella et al. 2025; Stewart-Yates et al. 2025). Among insects attracted to decomposing remains for feeding and oviposition or larviposition, Diptera blowflies (Calliphoridae) and flesh flies (Sarcophagidae) are typically the earliest colonizers after death and are frequently found in high abundance in almost all stages of decomposition (Smith 1986; Marcondes and Thyssen 2017). To date, more than 70 species of Calliphoridae and 950 species of Sarcophagidae have been recorded in the Neotropical region (Kosmann et al. 2013; Wolff and Kosmann 2016; Marinho et al. 2017; Pape 2021), with about 15 blowflies and 15 flesh flies of forensic importance in this region (Carvalho and Mello-Patiu 2008; Madeira-Ott et al. 2022). In advanced stages of decomposition, beetles become prevalent (Smith 1986), especially species belonging to Staphylinidae, Histeridae, Hydrophilidae, Scarabaeidae, Carabidae, Cleridae, and Dermestidae families (e.g., Corrêa et al. 2019; Gaedke et al. 2024). Staphylinidae, Histeridae, and Carabidae families include predatory species that can be found at the same decomposition stages as fly larvae, since they feed on them (Almeida and Mise 2009).

Considering the PIA reliably and accurately represents the PMI and the increasing use of entomological evidence in forensic cases (e.g., Oliveira-Costa and Mello-Patiu 2004; Pujol-Luz et al. 2006; Kosmann et al. 2011; Thyssen et al. 2012, 2018; Souza et al. 2014, 2026; Vasconcelos et al. 2014, 2019; Pérez et al. 2017; Corrêa et al. 2019; Lira et al. 2020a, 2020b; Amat et al. 2021; Ramos-Pastrana et al. 2021, 2022; Siri et al. 2021; Battán-Horenstein et al. 2023; Barros et al. 2024; Botteon et al. 2024a, 2024b; Gaedke et al. 2024), we report here a case in which *Hemilucilia segmentaria* (Fabricius) (Diptera, Calliphoridae) was used as entomological evidence to calculate the PMI in a suspected death of a woman whose body was found in a wild environment in southeastern Brazil. Additionally, we report the breeding of *Sarcophaga* (*Lyopygia*) *ruficornis* (Fabricius) (Diptera, Sarcophagidae) in the corpse, highlighting the need of data development of this species for accurately estimation of PMI, and *postmortem* lesions caused by the previous fly species and by the carrion beetle *Oxelytrum discicolle* (Brullé) (Coleoptera, Staphylinidae, Silphinae), emphasizing the importance of the knowledge about the biology and behaviour of necrophagous species in order to avoid misinterpretation of evidences.

## Material and methods

The oldest fly larvae, all in third instar, were collected from the corpse, on the head, behind the ears and inside the lesion observed on the neck, in two different moments for PMI estimation: the first one, in the scene; and the second one, at the Forensic Medical Institute, approximately eight hours after the first collection. Solely adult beetles were observed and collected from the corpse, on the head, arms, and belly dermis, only at the scene. The collected fly larvae were divided into two groups, one stored in vials containing 70% ethanol, and the other one, stored in vials with Kahle's solution (prepared as proposed by Borror et al. 1981), in order to have better material preservation for species determination, using morphological characters.

Fly larvae and adult beetles were observed with the aid of a Carl Zeiss™ Discovery V. 12 stereomicroscope with an Axiocam MRc5 image capture system and Zen Pro 2012™ software with depth focus for visualizing internal and external anatomical characters. To observe the cephaloskeleton of fly larvae, dissection was performed, and followed by clarification with a 10% potassium hydroxide (KOH) solution, as proposed by Sukontason et al. (2004). Keys and descriptions (Thyssen and Linhares 2007; Almeida and Mise 2009; Thyssen 2010; Grella 2016; Prado et al. 2023) were used for species determination. Fly larval length was measured using Zen Pro™ 2012 software, and body mass, with a Bel™ analytical balance (model MARK M214A, with a minimum sensitivity of 0.001 g).

Meteorological data (daily averages for temperature, humidity, and accumulated rainfall) were obtained from the closest meteorological station to the scene, about 7.5 km far from each other (CIIAGRO 2026).

## Results

### Case report

**Fig. 1:**
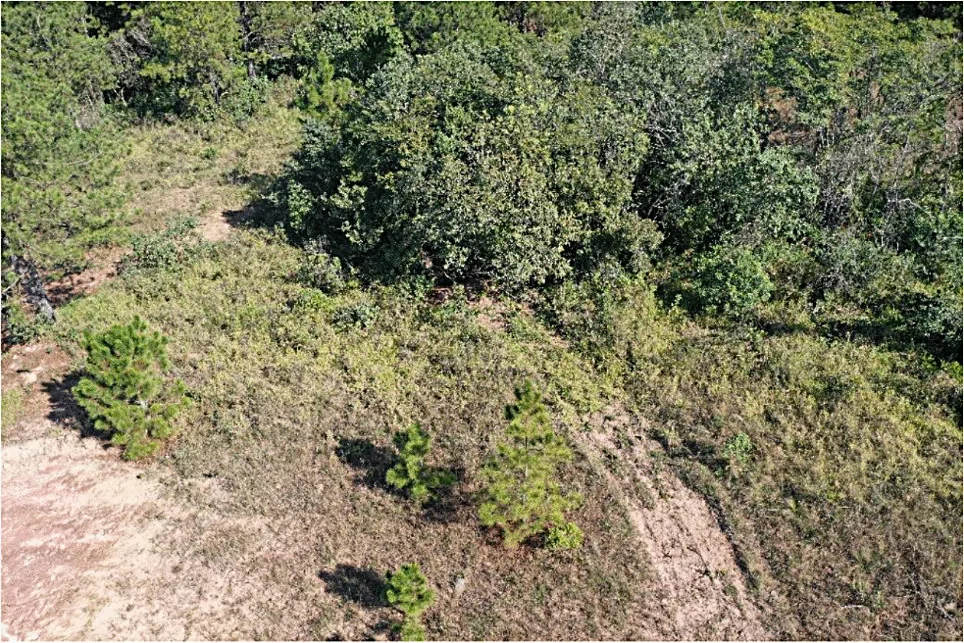
Panoramic view of the area where the corpse was found in Southeastern Brazil

**Fig. 2:**
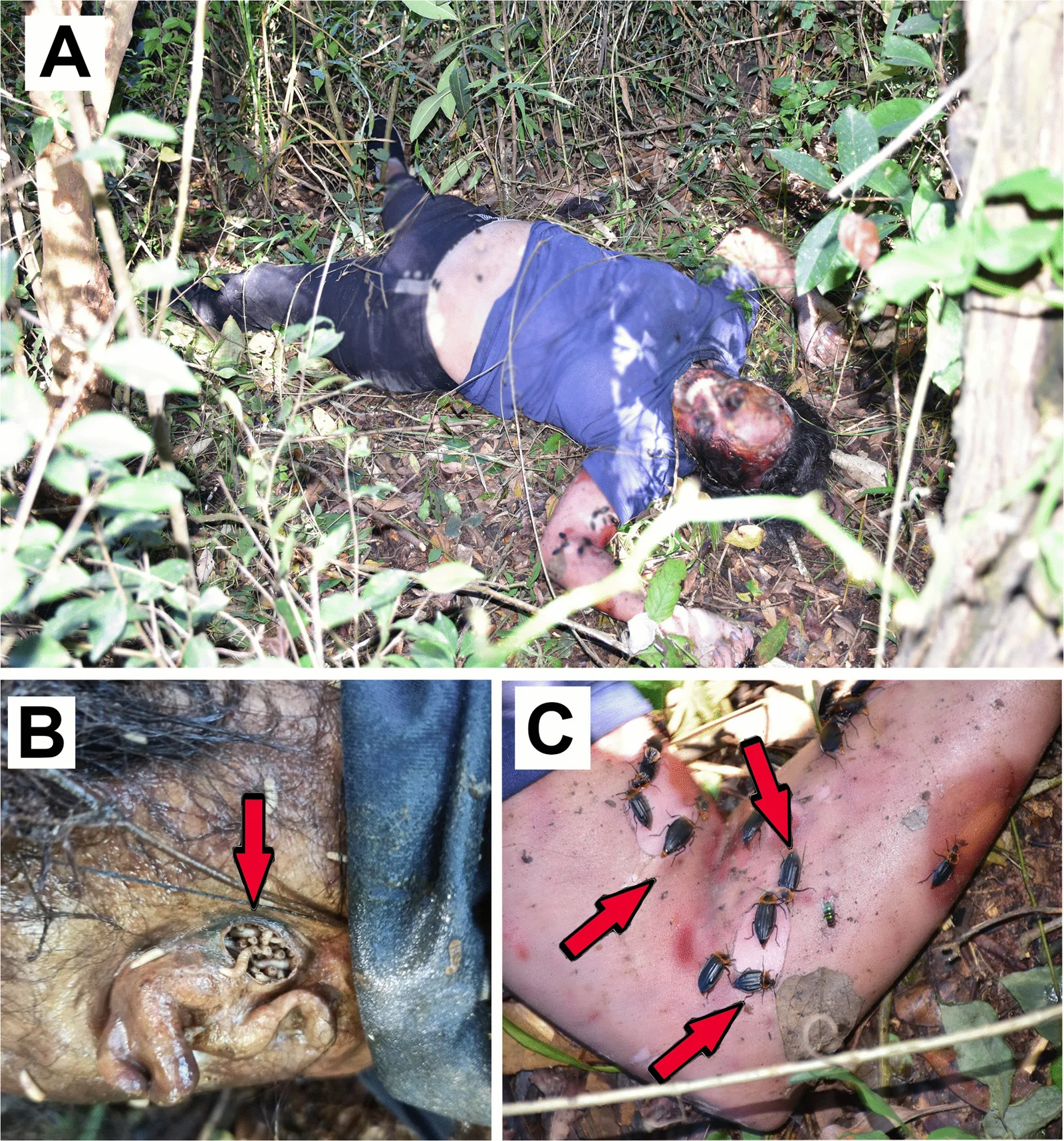
Crime scene. In: (**A**) the surroundings where the corpse was found; (**B**) victim's head in supine position; and, on the red arrow, “shotgun-like” injury caused by fly’s larvae on the victim’s hindhead; (**C**) *Oxelytrum discicolle* (Brullé) (Coleoptera, Staphylinidae, Silphinae) causing circular injuries on the dermis of the victim's left arm

**Fig. 3:**
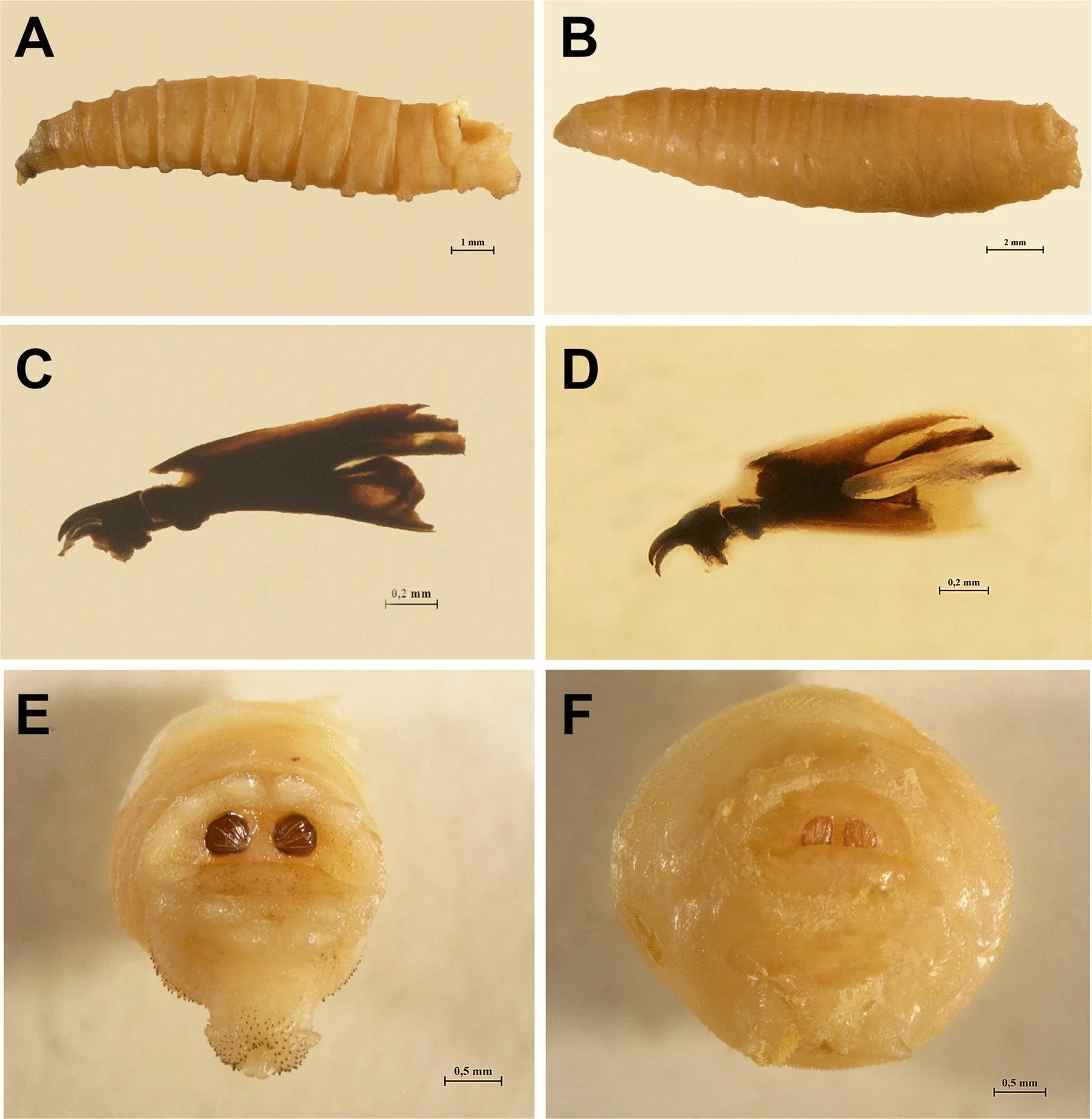
Third-instar larvae of *Hemilucilia segmentaria* (Fabricius) (Calliphoridae) (**A**; **C**; **E**) and *Sarcophaga* (*Lyopygia*) *ruficornis* (Fabricius) (Sarcophagidae) (**B**; **D**; **F**) collected from the corpse and their diagnostic characters. In: (**A**) and (**B**) *Habitus* in lateral left view; (**C**) and (**D**) cephaloskeleton, lateral left view; (**E**) and (**F**) anal division, posterior view

A middle-aged clothed woman’s corpse was found near a back road, in a wooded area (Fig. 1), in southeastern Brazil. The corpse was in a dorsal decubitus position and presented an intermediate decomposition stage, however with a significant difference among the head (more advanced) and the other body’s parts (less advanced). There was a large amount of fly larvae, especially in the head area, and many beetles throughout the body (Fig. 2). Third-instar larvae collected from the corpse were identified as belonging to the species *Hemilucilia segmentaria* and *Sarcophaga* (*Lyopygia*) *ruficornis* (Fig. 3). Collected beetles were identified as *Oxelytrum discicolle* (Fig. 2C).

During local examination, the forensic expert observed fly larvae and beetles in the corpse causing *postmortem* injuries, including circular injuries on the dermis of the victim’s left arm and a “gunshot-like” injury in the hindneck (Fig. 2B, red arrow). The forensic pathologist also confirmed these injuries as *postmortem* artifacts caused by insects’ activity, during the necropsy.

Meteorological data of the last 10 days before the corpse was found pointed out an average daily temperature of ca. 20.0°C, 50% of relative humidity, and 18.0 mm of accumulated rainfall (CIIAGRO 2026). The recorded average larval body mass was of 46.0 ± 0.1 mg for *H*. *segmentaria* (N = 10), which in comparison with the data retrieved from Thyssen (2005), available only for weight, allowed a PMI inference of 80 h (ca. three days), including egg incubation time. On average, body mass of *S.* (*L*.) *ruficornis* (N = 3) was 212.3 ± 34.1 mg with a length of approximately 19.0 ± 0.5 mm. Developmental data for *S.* (*L.*) *ruficornis* is only available for 25 °C (Nassu et al. 2014), considering body mass and length, which in comparison with our data returned an inference of 60 h. Considering the pattern occurring for other necrophagous species of Diptera (e.g. Campobasso et al. 2001; Thyssen 2005; Nassu et al. 2014; Vairo et al. 2015; Grella et al. 2025), that the time of development increases as the temperature decreases, we infer the developmental time for *S*. (*L*.) *ruficornis* at 20 °C is longer than 60 h.

Considering the age of the oldest insect (*H*. *segmentaria*), the PIA returned a minimum PMI of ca. three days. Despite the missing person report of 13 days, the PMI estimation based on the entomological evidence directed the subsequent police investigations, which found a record of security cameras of the victim walking in the surroundings of the area where the corpse was found four days before the fact. This video corroborated the PMI estimation close to the last time the victim was seen alive.

## Discussion

Although there are different methods to estimate the PMI (e.g., *rigor mortis*, *livor mortis*, liver temperature, among others; see López and Navadijo (2025) for a comprehensive review), the use of necrophagous insects becomes the most reliable biological approach when the body is in an advanced stage of decomposition (Catts and Goff 1992). However, the robustness of this approach depends directly on the quality of crime scene observation, collection, preservation, and identification of the entomological evidence (Catts 1992).

Even though the corpse was placed in a wooded area, the forensic expert’s report pointed out the victim’s head was more exposed to the sunlit in comparison to the rest of the body, which was in a completely shaded spot. Additionally, the victim was clothed and it is well documented that the decomposition process begins by insects’ colonization of the natural moist body orifices (Catts 1992). These facts explain why the victim’s head has the appearance of a more advanced decomposition stage in comparison with the rest of the body.

The availability of reliable ecological insect datasets for the region where the crime occurred is of paramount importance. The study by Thyssen (2005) provides thermal requirements for two Calliphoridae species, one of them is *H*. *segmentaria*, thereby supporting the applicability and reliability of entomological data in the present case.

Numerous studies have demonstrated the influence of temperature on the biological development of fly larvae (e.g. Thyssen 2005; Nassu et al. 2014; Lecheta et al. 2015) and beetles (e.g. Richardson and Goff 2001; Velásquez and Viloria 2009; Wang et al. 2016; Lira et al. 2020b), where differences of as little as 3.0–4.0°C are sufficient to either delay or accelerate development. For *S*. (*L*.) *ruficornis*, developmental data are available only for a single temperature (25.0°C; see Nassu et al. 2014), which is 5.0°C higher than the mean temperature recorded at the crime scene. This discrepancy renders PMI estimates based on this species less reliable. Also, it highlights the need for more bionomic studies for supplying a reliable dataset of species development for application in real cases.

The beetle species found at the crime scene, *O*. *discicolle*, is frequently reported in forensic studies (Almeida et al. 2015; Lira and Vasconcelos 2016) and has already been used for PMI estimation (Lira et al. 2020b), highlighting its forensic relevance. As discussed by Oliva and Di Iorio (2008), adults of *O*. *discicolle* are omnivorous, whereas larvae are obligately necrophagous. Adults are attracted to carcasses in advanced stages of decomposition in both natural and urban environments (Lira and Vasconcelos 2016; Costa-Silva et al. 2017; Lira et al. 2019).

According to the forensic expert’s report following the perinecroscopic examination, no larvae of *O*. *discicolle* were found, despite the considerable number of adults present at the crime scene (see Fig. 1). Based on the data presented by Velásquez and Viloria (2009), the minimum duration (in days) of the developmental stages of *O*. *discicolle* at 20.0°C is 2.9 days for the egg stage and 2.0 days for the first larval instar (L1). Considering these developmental parameters, L1 would be expected to appear only after approximately four days *postmortem*. However, according to the PMI estimated in the present study, based on the developmental biology of *H*. *segmentaria*, the victim had been dead for only three days. This discrepancy may explain the absence of *O*. *discicolle* larvae.

Knowledge of the biology, behavior, and natural history of necrophagous species is essential for the proper interpretation of entomological evidence in criminal cases. Ururahy-Rodrigues et al. (2008), for example, describe the displacement of a pig carcass caused by the beetle *Coprophanaeus* (*Megaphanaeus*) *lancifer* (Linnaeus) (Coleoptera: Scarabaeidae). Vertebrates may also alter a crime scene. Lira et al. (2020a), for instance, demonstrate how necrophagous vertebrates can interfere with carcass chronology, while Souza et al. (2026) report a homicide case in southeastern Brazil in which the victim’s leg was removed due to vertebrate scavenging activity.

Necrophagous fly larval activity was responsible for producing an artifact, a “gunshot” like pattern injury in the victim's hind head (Fig. 2B, red arrow). Similarly, adults of *O*. *discicolle* were observed feeding on the body, creating circular lesion patterns visible on the victim’s dermis (Fig. 2C). Identification of such lesions is intrinsically linked to the behavioral ecology of *O*. *discicolle* as well as to larval fly feeding. Without this knowledge, these lesions could be misinterpreted as *antemortem* injuries, potentially leading to erroneous investigative conclusions.

Although the studies by Thyssen (2005) and Velásquez and Viloria (2009) support the application of entomological data in the present case, the lack of developmental data for other species highlights significant knowledge gaps in forensic entomology. It is expected that this case report will encourage further research on taxonomy (e.g., identification keys), behavior, and developmental biology of forensically relevant species at both national and global scales, ultimately ensuring the availability of robust datasets for use in criminal investigations involving suspected or violent deaths.

## Data Availability

The datasets generated and analyzed during the current study are available from the corresponding author on reasonable request.
